# Moderate Beer Consumption Is Associated with Good Physical and Mental Health Status and Increased Social Support

**DOI:** 10.3390/nu15061519

**Published:** 2023-03-21

**Authors:** Antonio Moreno-Llamas, Ernesto De la Cruz-Sánchez

**Affiliations:** Public Health and Epidemiology Research Group, San Javier Campus, University of Murcia, 30720 San Javier, Spain

**Keywords:** alcohol, beer, mental health, social support, daily functioning, public health

## Abstract

There is little large-scale evidence on the effect of alcoholic beer consumption on physical, mental and, above all, socio-emotional health. Here, we conducted a secondary data analysis of the 2012 and 2017 National Health Surveys with 33,185 individuals aged 18 years and older to assess beer consumption in relation to self-perceived health, functional limitations, mental health, and social support. Logistic regression models assessed the association of alcohol consumption (abstainers, ex-drinkers, occasional drinkers, moderate beer drinkers, and heavy beer drinkers) with self-perceived health (poor or good), limitations of type (none, physical, mental, or both) and intensity (none, mild, or severe), mental health (poor, average, or good) and social support (poor, average, or good). Analyses were adjusted for sex, age, occupational social class, educational level, place of residence, survey, part-time physical activity, dietary information, smoking, and body mass index. Compared to abstainers, occasional and moderate beer drinkers were associated with better mental and self-perceived health and social support, and were less likely to report mild or severe physical limitations. In contrast, former drinkers were associated with worse indicators of self-perceived health, physical health, mental health, and social support than abstainers. Alcoholic beer consumption showed a J-shaped relationship with self-perceived, physical, mental, and social-emotional health, with better values at moderate levels.

## 1. Introduction

A large body of observational epidemiological studies has suggested that alcohol doses of 1–2 standard units of drink (10 g of alcohol per drink) for men and between 0 and 1 standard unit of drink for women per day may reduce the risk of all-cause mortality due to improved cardiovascular disease mortality [1,2,3,4]. Despite decades of evidence, the famous J-shaped relationship between alcohol consumption and human health is still a matter of debate [1,5,6,7,8,9,10,11,12]. The complex relationship of alcohol consumption with health has been reported to be beneficial at low doses and, conversely, to have deleterious effects at high doses compared to non-drinkers. However, recent, more robust studies have shown that the benefits of alcohol consumption may have been overestimated, or even negligible in some cases. [1,11,12,13]. Moreover, as cited in the 2016 Alcohol Global Burden Disease, *the only safe dose of alcohol is zero* [10]. Unexpectedly, the Alcohol Global Burden Disease 2020 study recently updated the epidemiological evidence in 2022 with new results that keep the old debate open [7]. This report maintains that the safest dose of alcohol for those under 40 is zero, but has found modest benefits in those over 40 for cardiovascular disease, diabetes, and stroke, regardless of sex, at amounts of 0.114–1.870 standard units of alcoholic beverage per day [7]. The authors have also highlighted the importance of the characteristics and distribution of a given region or population in the complex relationship between alcohol consumption and health status [14]. While these observational studies remain inconclusive, on the other hand, the experimental literature has strongly pointed to the benefits of light to moderate alcohol consumption and the harmful properties of excessive consumption, also focusing on specific types of alcoholic beverages such as wine or beer [4,15,16,17,18,19,20,21].

Beer—the most widely consumed beverage in the world—is a relatively low-alcohol beverage (5%) that influences a wide range of health aspects such as the cardiovascular system, the immune system, osseus tissue, redox status, the nervous system, and mental illness [9,20,21,22,23]. In this regard, the health effects of moderate alcohol consumption have been attributed to changes in blood lipid profiles, anti-inflammatory, antioxidant and even anti-cancer effects on immune system function, improved insulin resistance, and reductions in physiological stress levels as measured by the hormones cortisol and ACTH [16,19,21,24]. In addition to these alcohol-related aspects, beer also has its own specific non-alcoholic components, including minerals (phosphorus, magnesium, potassium), vitamins (B group), and polyphenols such as xanthohumol from hops, with antioxidant, anti-cancer, anti-inflammatory, and anti-viral properties [2,9,20,22,23], or the reduction of the risk of osteoporosis by increasing bone mineral density [9,22]. 

However, other non-physiological domains may come into play in the complex health relationship to elucidate the effects of alcohol and beer beyond physical health, such as socio-emotional health, mental health, and well-being. Socio-emotional and mental health are closely related not only to each other, but also to physical health in the development of non-communicable diseases and premature mortality from all causes [25]. Social isolation—the self-perceived discrepancy between actual and desired social networks—has recently been considered to be of great public health concern and importance, especially in older adults in high-income and ageing countries [25]. Social deprivation may be associated with harmful lifestyle behaviors and, vice versa, strong social networks, both in quantity and quality, may favor less harmful habits, such as moderate alcohol consumption [26]. Similarly, people’s alcohol consumption and drinking patterns are rooted in contextual and cultural factors: northern European countries, for example, tend to consume alcohol outside of meals at home, whereas in Mediterranean countries such as Spain, alcohol consumption, and especially beer consumption, involves a large social context within meals and a more moderate intake [27]. The latter, in turn, could be beneficial not only for mental health, but also for physical and emotional health, going some way to explaining the possible health effects of alcohol consumption in people over 40 (in whom social isolation is more prevalent), as reported in the Alcohol Global Burden Disease 2020 report [7]. However, there is little large-scale quantitative evidence on this emerging issue in relation to alcoholic beer consumption and physical, mental, and especially socio-emotional health.

Therefore, we assessed the associations of beer consumption (a low-dose, widely consumed alcoholic beverage) with self-perceived health, daily functioning, mental health, and social support using data from two Spanish National Health Surveys (2012 and 2017).

## 2. Materials and Methods

The present study was carried out according to the STROBE (STrengthening the Reporting of OBservational studies in Epidemiology) Statement for cross-sectional studies [28].

### 2.1. Data

We performed a secondary data analysis employing cross-sectional data from two waves of the Spanish National Health Surveys (SNHS) in 2012 (*n* = 21,007) and 2017 (*n* = 23,089) in which the Spanish population 15 years and over was surveyed about health status, determinants of health (e.g., health-related lifestyle variables or preventive health care use), and diverse sociodemographic domains. Both cross-sectional surveys were conducted by the Spanish Ministry of Health, Consumption and Social Welfare and the Spanish National Statistics Institute using a multistage stratified sample strategy to draw potential surveyed participants. The multistage sampling selection method comprised three stages with a proportional random probability in which census units, household dwellings, and finally participants were sequentially drawn based on age and gender population representativeness. Only one individual was randomly selected per sampled household. Trained interviewers conducted face-to-face interviews at participants’ households. The information collection comprised four 15-day periods to avoid possible biases related to seasonal health-related lifestyle behaviors, morbidity, and other health indicators. The response rates for 2012 and 2017 SNHS were 89.6% and 74.0%, respectively. More detailed information about SNHS is published elsewhere [29].

### 2.2. Alcohol and Beer Consumption

Alcohol and beer consumption was self-reported by participants who were surveyed about their alcohol drinking frequency during the last 12 months with 9 possible answers: *Daily or almost daily*, *5*–*6 days per week*, *3–4 days per week*, *1–2 days per week*, *2–3 days in a month*, *Once a month*, *Less than once a month*, *Not in the last 12 months because I have stopped drinking alcohol*, and *Never or only a few sips in my life*. Those people who responded *2–3 days in a month*, *Once a month*, *Less than once a month* were treated as *Occasional* drinkers because additional information regarding type and quantity of alcoholic beverages consumption was not provided in the surveys. Furthermore, those who reported that they have not *drunk alcohol in the last 12 months because I have stopped* were classified as *Ex-drinkers*, while those who reported *never or only a few sips in my life* were denominated *Abstainers* or *Non-drinkers*. The remained groups (*Daily or almost daily*, *5–6 days per week*, *3–4 days per week*, *1–2 days per week*) were additionally interviewed about the number and type of alcoholic drinks each day of the week. The surveys included six types of alcoholic drinks: beer, wine or cava, vermouth, liqueurs, whisky or mixed drinks, and local beverages. The equivalences (in grams of pure alcohol) proportionated by the SNHS were as follows: beer (10 g per unit of drink), wine or cava (10 g per unit of drink), vermouth (20 g per unit of drink), liqueurs (20 g per unit of drink), whisky or mixed drinks (20 g per unit of drink), and local beverages (10 g per unit of drink). Thereby, we computed the weekly total and type-specific alcohol consumption in grams of pure alcohol and then determined the type of drinkers based on previous studies [30,31,32]. A predominant beer drinker was defined as when the percentage of alcohol consumed from beer out the total alcohol intake was more than half [30,31,32]. When drinkers did not present a predominant type of alcohol consumption, they were classified as mixed drinkers. 

In this study, we excluded non-specific beer and mixed drinkers (*n* = 9602; 21.78%). We also removed those participants with incomplete missing information regarding alcohol consumption (*n* = 469; 1.06%). Our study only included all those who had reported being abstainers from alcohol consumption, ex-drinkers (no alcohol consumption in the past 12 months), occasional alcohol drinkers, moderate beer drinkers, and high beer drinkers. Due to the low frequency of consumption in occasional drinkers, the SNHS did not continue surveying the frequency and type of alcoholic beverages of total weekly alcohol consumption. Alcoholic beer drinkers were finally classified as Occasionally (previously described), Moderate (Women: up to 12 g of alcohol per day, Men: up to 24 g of alcohol per day) or High (Women: more than 12 g of alcohol per day, Men: more than 24 g of alcohol per day) according to previous studies and alcohol guidelines for the hypothetical detrimental effect of alcohol use [1,2,3,22,23,31], along with abstainers and ex-drinkers. The moderate and high alcohol thresholds were set using the total amount of alcohol and not exclusively those from alcoholic beer.

### 2.3. Health and Social Support Variables

Health status was measured by self-assessment of health status, long-term medical problems, limitations due to health problems, mental health symptoms, and social support. Self-rated health status was described by participants using a 5-Likert-Scale: *In the last twelve months, would you say that your state of health has been very good, good, fair, bad, or very bad?* The self-rated health status was then dichotomized into *Good* (Very Good and Good) and *Poor* (Very Bad, Bad, and Fair). Participants were also asked for any limitation due to a health problem over at least the last 6 months in performing activities (*Not limited*, *Limited but not severely*, or *Severely limited*), and about what type of problem was the cause of that difficulties, grouped into *Physical*, *Mental*, *Both*, or *None*. Mental health symptoms were measured employing the General Health Questionnaire (GHQ-12) to obtain a composite index by means of 12 mental health-related questions about participants’ mistrust, tension, overwhelmed, incapable, unhappy, and general problems, among others, in 4-Likert-Scales (e.g., *Not at all*, *No more than usual*, *Somewhat more than usual*, or *Much more than usual*) [33]. To compute the final composite mental health symptoms index, the first two possible answers were scored as 0 (i.e., *not at all*, *no more than usual*), and the last two (i.e., *somewhat more than usual* or *much more than usual*) as 1 [33]. We then summed the 12 answers, and thereby, the final index ranged from 0 (better mental health) to 12 (poorer mental health). Due to the highly skewed non-normal distribution with a high number of zeros, the mental symptoms index was categorized into three groups as follows: *Good* (0–3), *Medium* (4–8), and *Poor* (9–12). The Cronbach’s alpha of mental health questions was excellent (0.902). Social support was also measured using the Duke–UNC Functional Social Support Questionnaire [34] with a composite index by means of 11 affective and personal support-related questions of daily life in a 5-Likert-Scale (from *Much less than I would like* to *As much as I would like*). The responses of the Likert scale were recoded from 1 to 5 and were then summed to obtain the index, which ranged from 11 (poor social support) to 55 (good social support). As in mental health symptoms index, the social support index was categorized into three groups: *Poor* (11–25), *Medium* (26–40), or *Good* (41–55). The Cronbach’s alpha of social support questions was excellent (0.907). 

### 2.4. Health-Related Lifestyle Variables

Among the main known health-related lifestyle variables, we used leisure-time physical activity, dietary information, and tobacco use. The body mass index was also included in this section, although it is not a lifestyle aspect, but a health risk factor arising from physical activity and diet patterns, among other causes. Leisure-time physical activity was assessed by the frequency with which participants do any physical activity or sport in their free time by asking, *Which of these possibilities best describes the frequency with which you do some leisure-time physical activity?* The four options were Sedentary (reading, watching TV, going to the movies and so on), Occasional (walking or cycling, gardening, and recreational activities that require light exertion), Several times a month (sports, gymnastics, running, swimming, cycling, or team games, among others), or Several times a week. The examples in the brackets of occasional, several times a month, and several times a week of leisure-time physical activity could belong to any of these groups, but the classification was only based on the frequency and not on the volume, intensity, or type of physical activity. Dietary information comprised fruit, vegetables, sweets, sweetened beverages, snacks, and fast-food consumption. Each dietary variable was measured by the frequency of consumption in five groups: Almost never or never, Less than once per week, Once or twice per week, Three or more times per week but not daily, or Once or more times per day. Tobacco use was also self-reported and grouped as Current smoker, Ex-smoker, or Non-(never-) smoker. Body mass index was computed from self-reported individuals’ height and weight, which was then classified according to the World Health Organization cutoff-points [35] into Insufficient weight (<18.5 kg/m^2^), Normal weight (18.5 to 24.9 kg/m^2^), Overweight (25 kg/m^2^ to 29.9 kg/m^2^), and Obesity (≥30 kg/m^2^).

### 2.5. Sociodemographic Variables

We employed several sociodemographic indicators as covariates (conjointly with health-related lifestyle variables) to control possible influences and/or differences in the relationship between alcohol use and health outcomes by gender (*Women* and *Men*), age, educational attainment, labor occupation, and size of the resident community. Age was used in all statistical analysis as categorical variable in three groups (*18–34, 35–64, ≥65 years old*). We also reported descriptive statistics using age as continuous variable. We restricted the studied population of the survey to those aged 18 and over (*n* = 998; 2.26%), the legal limit for buying and drinking alcohol in Spain. Regarding educational attainment, respondents self-reported their highest completed educational level among 24 options in the original raw data, from which SNHS establishes eight groups as follows: *Cannot read or write, Incomplete primary education (attended less than 5 years of school), Primary education complete, First stage of Secondary Education with or without diploma, Bachelor studies, Intermediate vocational education or equivalent, Higher vocational education or equivalent, University studies or equivalent*. We finally regrouped educational attainment to only three categories (*No studies or primary education, Secondary studies, University studies*). Thus, *no studies or primary education* included cannot read or write, incomplete primary education (attended less than 5 years of school), and primary education complete; *secondary studies* included first stage of secondary education with or without diploma, bachelor studies, intermediate vocational education or equivalent, and higher vocational education or equivalent; and university studies included university studies or equivalent categories [36]. The labor occupation constituted the occupational social class based on the type of occupation of the main breadwinner of the household. The occupational social class was categorized into I-II, High social class (executives of government and companies, senior civil servants, professionals, technicians, managers and owner–managers of commerce and personal services, other non-high technicians, artists, and athletes); III, Middle social class (middle managers, administrative personnel, military protection, and security services), and IV–V, Low social class (semiskilled and manual workers in industry, commerce and services, and unskilled workers) according to previous studies and the Working Group on Determinants of Health of the Spanish Society of Epidemiology [37,38]. The reduction of responses of the educational attainment and occupational social class into three groups was done in order to ensure an appropriate sample size per group according to the International Standards Classification of Education and international European reports in the use of socioeconomic status variables to facilitate statistical analyses and the interpretation of the findings [39]. The size of the resident community (*Resident place* hereinafter) was based on the number of inhabitants of the municipality where participants resided. Further, resident place was classified into *Metropolitan* places (more than 500,000 inhabitants), *Suburban* places (10,000 to 500,000 inhabitants), and *Rural* places (less than 10,000 inhabitants).

### 2.6. Statistical Analysis

First, we computed descriptive statistics in continuous (mean ± standard deviation) and categorical variables (sample size and percentages) to show the characteristics of the overall sample and across drinking groups among abstainers, ex-drinkers, occasional drinkers, moderate beer drinkers, and high beer drinkers. Overall differences among drinking and information groups were analyzed by chi-squared and one-way between-subject ANOVA analyses. Second, binomial and multinomial logistic regressions were run to assess the association between drinking beer consumption (independent variable) and self-rated, physical, and mental health status as well as social support, adjusting by gender, age groups (18–34, 35–64, ≥65), occupational social class, educational attainment, resident place, survey, leisure-time physical activity, fruit, vegetables, sweets, sweetened beverages, fast-food and snacks intake, tobacco use, and body mass index. Additionally, we performed some sensitive analyses to test the robustness of the findings. First, we reran the logistic regression models, excluding those who had experienced binge drinking in one day (at least 5 units of standard drinks) in the last 12 months; second, we segmented the analyses by gender (women and men); and third, by age (18–39 and ≥40). Figures for segmented and sensitivity analyses are provided in the Supplementary Materials (Figures S1–S15). Odds ratio (OR) and confidence intervals at 95% (95%CI) were calculated in logistic regression models. Group references in the logistic regression models were women, aged 18–34, of low social class, primary or without studies education, survey year 2012, rural resident, sedentary, non-(never-)smoker, normal weight, and almost never or never dietary information. Statistically significant differences were established at *p* < 0.05. All statistical analyses and data handling were performed using Rstudio version 3.6.1 (Rstudio, Inc., Boston, MA, USA), and binomial and multinomial logistic regressions were conducted with *glm* and *multinom* functions from the packages *stats* and *nnet*, respectively.

## 3. Results

### 3.1. Descriptive Results

General sample characteristics results are provided in the Supplementary Material (Table S1). The total sample comprised 43,098 adults, among which there were higher proportions of women, those aged 35–64 years, and those with secondary education, followed by primary education, low occupational social class, residing in suburban areas. This sample was slightly larger in the 2017 SNHS. Regarding health-related lifestyle variables, 41.79% of the general population reported being sedentary, and only 38.11% engaged in occasional physical activity in their leisure time. The daily consumption of fruit and vegetables was 66.30% and 44.71%, respectively, while the frequency of sweets consumption presented more dispersion. Furthermore, more than half of the respondents were overweight or obese (56.24%), and smokers represented a quarter of the population (24.67%). In health variables, two thirds (66.52%) of the population considered themselves as having good health status, 73.54% had no limitations in their daily life, and 21.40% described physical limitations with only 5.06% reporting severe limitations. More than 85% had good mental health and social support. Finally, regarding alcohol consumption, 20.89% were abstainers, 14.84% were ex-drinkers, and 26.69% occasionally consumed alcohol. Moderate beer consumption accounted for 30.62% and high drinking for only 6.98% of the studied population. Among the different types of beverages, beer (15.16%), wine (13.48%), and mixed (5.34%) consumption predominated. Additionally, more than half had never had acute binge alcohol episodes (53.68%). 

On the other hand, when the results were differentiated according to beer consumption groups in Table 1 (abstainers, ex-drinkers, occasional, moderate, and high drinkers), men were predominantly moderate and high beer drinkers, whereas, in contrast, women were generally abstainers, ex-drinkers, and occasional drinkers. Across age, people older than 65 years were found to have a lower proportion of high beer consumption (i.e., the older the age, the lower the alcohol consumption), whereas the 35–64 age group had a higher proportion of high beer consumption. Occasional, moderate, and high drinkers were also more educated, with university and secondary education, and belonged to the middle and upper occupational social classes. The corresponding survey to 2017 contained a higher proportion of occasional, moderate, high, and ex-drinkers. Regarding place of residence, the proportion of metropolitan residents increased with higher beer consumption, while rural and suburban residents slightly varied across groups. In terms of health-related lifestyle behaviors, those who reported occasional, moderate, or high consumptions were less sedentary and more physically active several times a week in their leisure time. Daily fruit and vegetable consumption decreases across alcohol consumption groups, mainly in high drinkers. Abstainers and ex-drinkers, on the other hand, had higher prevalence than the other groups in daily consumption of fruit and vegetables. Daily consumption of sweets, soft drinks, snacks, and fast food were also higher in high drinkers, where abstainers and ex-drinkers showed lower intakes. As for the body mass index, occasional drinkers had a higher proportion of normal weight, moderate drinkers had a higher proportion of overweight but lower for obesity, and abstainers and ex-drinkers described the highest proportion of obesity. Moreover, the proportion of current smokers was higher along with a higher alcohol consumption.

**Figure 1:**
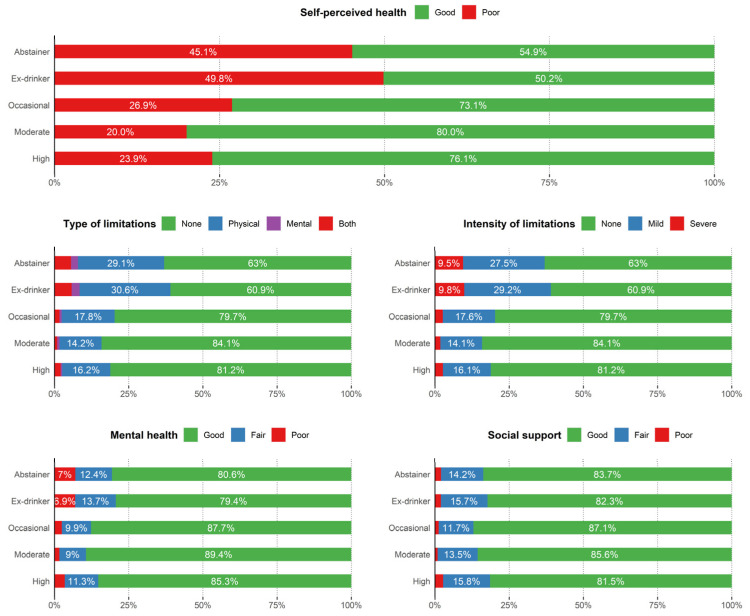
Percentages of self-perceived health, type, and intensity of daily functional limitations, mental health and social-affective support in the Spanish adult population (≥18 years) according to beer consumption. Alcoholic beer drinkers were finally classified as occasional (a maximum of 3 days per month), moderate (women: up to 12 g of alcohol per day; men: up to 24 g of alcohol per day) or high (women: more than 12 g of alcohol per day; men: more than 24 g of alcohol per day). National Health Survey of Spain 2012, 2017.

On health-related variables (Figure 1), the best self-perceived health was observed in moderate beer consumption, followed by occasional and high drinkers. Ex-drinkers presented the highest prevalence of poor self-perceived health status. This same pattern was repeated for all other indicators of physical and mental health, and social support in which moderate and occasional drinkers described the best health (self-perceived health, daily limitations in type and intensity and mental health) and social support values, while ex-drinkers the worst. Abstainers reported worse health indices and social support than drinkers. The lowest prevalence of disease (physical, mental, or both) was observed in moderate and high drinkers, while the highest was in abstainers and ex-drinkers. The highest limitations (both mild and severe) were also reported in abstainers and ex-drinkers. Finally, mental health was better in moderate and occasional drinkers, as well as in social support, with worse results in ex-drinkers and abstainers, followed by heavy drinkers.

### 3.2. General Logistic Regression Model Results and Sensitive Analyses

**Figure 2:**
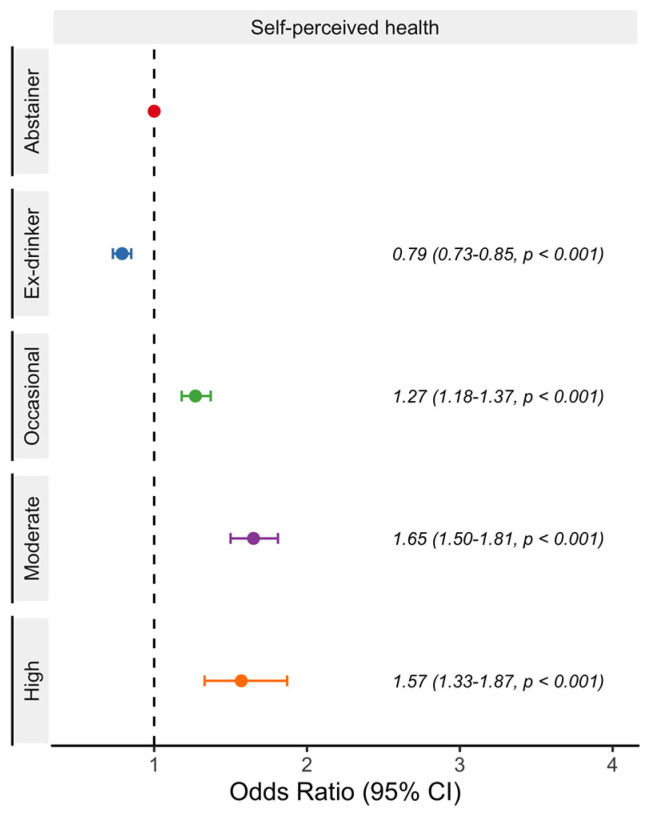
Odds ratio and 95% confidence intervals of reporting good self-perceived health in the Spanish adult population (≥18 years) according to beer consumption. Drinkers were classified as occasional (a maximum of 3 days per month), moderate (women: up to 12 g of alcohol per day; men: up to 24 g of alcohol per day), or high (women: more than 12 g of alcohol per day; men: more than 24 g of alcohol per day). Analyses adjusted for age, gender, social class, educational level, year of the survey, place of residence, physical activity, diet, tobacco, and body mass index. National Health Survey of Spain 2012, 2017.

**Figure 3:**
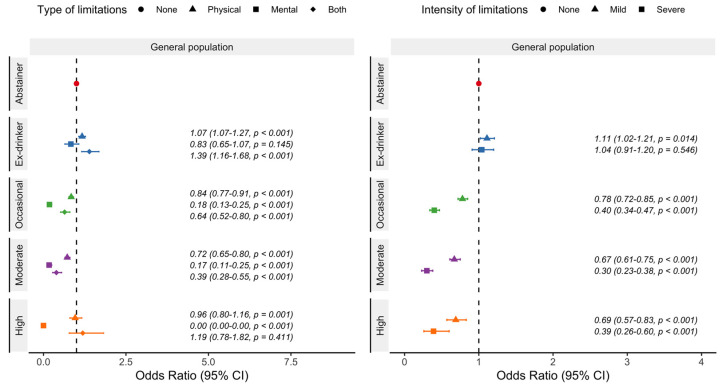
Odds ratio and 95% confidence intervals of reporting daily physical, mental, or both limitations and daily mild or severe limitations in the Spanish adult population (≥18 years) according to beer consumption. Drinkers were classified as occasional (a maximum of 3 days per month), moderate (women: up to 12 g of alcohol per day; men: up to 24 g of alcohol per day), or high (women: more than 12 g of alcohol per day; men: more than 24 g of alcohol per day). Analyses adjusted for age, gender, social class, educational level, year of the survey, place of residence, physical activity, diet, tobacco, and body mass index. National Health Survey of Spain 2012, 2017.

Logistic regression model analyses showed that, compared to abstainers, ex-drinkers were less likely to report a good self-perceived health. Remarkably, a J-shape relationship with better self-perceived health outcomes was observed for moderate beer consumption (Figure 2). Occasional, moderate, and even high drinkers were more likely to report good self-perceived health than abstainers. On the other hand, regarding type and intensity of limitations, ex-drinkers were more likely to have mild but not severe limitations (Figure 3). Conversely, occasional, moderate, and high drinkers were less likely to have mild and severe limitations. By type of limitation, ex-drinkers were more likely to report physical illness. Occasional, and moderate drinkers were less likely to report physical, mental, and both type of limitations (Figure 3). High drinkers were less likely to report physical and both limitations. 

**Figure 4:**
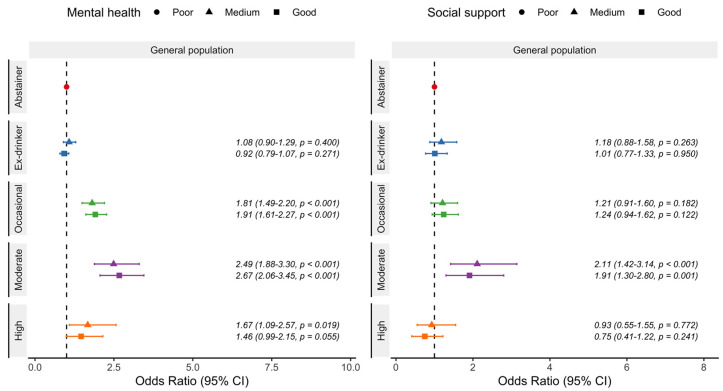
Odds ratio and 95% confidence intervals of reporting medium and good mental health and social support in the Spanish adult population (≥18 years) according to beer consumption. Drinkers were classified as occasional (a maximum of 3 days per month), moderate (women: up to 12 g of alcohol per day; men: up to 24 g of alcohol per day) or high (women: more than 12 g of alcohol per day; men: more than 24 g of alcohol per day). Analyses adjusted for age, gender, social class, educational level, year of the survey, place of residence, physical activity, diet, tobacco, and body mass index. National Health Survey of Spain 2012, 2017.

In mental health (Figure 4), occasional, and moderate drinkers were more likely to describe medium and good mental health compared with those who reported poor mental health scores in a J-shape relationship. High drinkers only presented higher odds of describing medium mental health. Occasional and moderate consumption were also more likely to report medium and good mental health in those who had never experienced binge drinking. Ex-drinkers showed no differences in the odds for medium nor good mental health compared with abstainers. Finally, regarding social support (Figure 4), only moderate drinkers were more likely to present medium and good social support scores. Occasional and moderate consumption were also more likely to report medium and good social support among those drinkers who had never experienced binge alcohol episodes. Ex-drinkers and high drinkers showed no differences for the odds of medium and good social support in comparison to abstainers. 

### 3.3. Gender and Age Logistic Regression Model Results

When logistic regression models were segmented by gender, both women and men showed the J-shape relationship in the self-perceived health where occasional, moderate, and even high drinkers were more likely to report a higher self-perceived health status compared with abstainers, whereas, on the contrary, ex-drinkers were more likely to report a poorer self-perceived health (Figure S2). There were slight gender differences in the associations of the intensity of daily limitations (Figure S8). Occasional and moderate women drinkers were less likely to report mild and severe limitations, and on the other hand, the reduced odds of mild and severe limitations among men were observed at any alcohol quantity (occasional, moderate, and high). Attending to the type of these limitations, the same results were found between women and men (Figure S5). Women were less likely to describe physical- and both-related (physical and mental) limitations at occasional and moderate consumption, and less likely to report mental-related limitations at occasional consumption. Men were less likely to describe physical-related limitations at moderate and high consumption, less mental-related limitations at any consumption (occasional, moderate, and high), and both-related limitations at occasional and moderate consumption. The relationship between beer consumption and mental health was similar in women and men. Both women and men were more likely to report medium and good mental health, compared with worse mental health, at occasional and moderate consumption (Figure S11). Men were also more likely to describe medium mental health among those occasional drinkers. On social support, women were more likely to report medium and good social support at moderate consumption; however, only men were more likely to report medium social support at this moderate level compared with worse social support (Figure S14). 

Attending to age groups (18–39 and ≥40), the younger population group was more likely to describe a better self-perceived health status at occasional and moderate levels, and the older population was even more likely to report the same at any consumption level (Figure S3). On the intensity of limitations, the younger group was less likely to report severe limitations at occasional, moderate, and high consumption but not for mild limitations (Figure S9). The older group was additionally less likely to describe both mild and severe limitations at any consumption level. By the type of limitations, the age differences were mainly observed at physical-related limitations (Figure S6). Whereas the younger group was not associated with physical-related limitations, conversely, the older group was less likely to report physical-related limitations at any consumption level. For mental health, the younger group was more likely to report medium, but not good, mental health at occasional and moderate consumption compared with worse mental health (Figure S12). Additionally, the older group was more likely to describe good mental health at any consumption level in comparison with those who reported worse mental health. Finally, the older group was more likely to report medium and good social support at moderate consumption compared with worse social support, but not for the younger group, for which no associations were observed (Supplementary Figure S15).

## 4. Discussion

### 4.1. Main Findings

Our results showed that moderate beer consumption may imply associations with better self-perceived, physical, mental and socio-affective health. Furthermore, our study replicated the J-shaped relationship between health and beer consumption in which moderate beer drinkers (women: 1 standard unit of drink per day; men: 2 standard units of drink per day) described the healthiest state even after controlling for health-related lifestyle behaviors (smoking, diet, and physical activity), BMI, socioeconomic status, and demographic factors. Compared to abstainers, drinkers who reported occasional alcohol consumption or moderate beer consumption were more likely to report higher self-perceived health, mental health, and social support, while they were also less likely to report mild and severe limitations and physical and/or mental limitations. Conversely, former drinkers were more likely to describe worse indicators of self-perceived health, daily limitations of type and intensity, mental health, and social support. The J-shaped relationship between beer and health was observed in both women and men, although women showed associations with better health status at lower doses than men. This health improvement with moderate doses of beer was observed mostly in drinkers aged 40 years and older, while little or no association was found among those under 40 years of age. Sensitivity analyses without those reporting severe acute drinking episodes showed the same results.

### 4.2. Comparisons with Other Studies and Potential Hypothetical Explanations

Previous empirical evidence is consistent with our findings. Regarding the relationship of health with alcohol consumption, in our case beer, several meta-analyses have investigated and re-analyzed the effects of alcohol consumption and drinking patterns on, primarily, physical health in terms of mortality and morbidity [2,4,6,11,40]. In summary, although there is a large body of research, the evidence remains uncertain. The use of reference groups such as abstainers alongside ex-drinkers and occasional alongside moderate drinkers has generated controversy in contributing to the famous J-shaped (or U-shaped) alcohol–health relationship, as other medium and large-scale studies with more robust and comprehensive methodological designs have found only detrimental health consequences [11,12,13]. However, the updated Global Burden of Disease review added that there may be benefits without gender differences for people over 40 years of age who consume between 0.114 and 1.870 standard units of drink daily, i.e., a moderate drinking habit [7]. In our study, we observed an improvement in health status with moderate doses of beer in drinkers aged 40 years and older and little or no association among those under 40 years. In addition, a randomized Mendelian study from the UK Biobank found that alcohol consumption of up to 17 standard drinking units per week (i.e., 2.43 standard drinking units per day) does not lead to premature ageing or death from telomere shortening [41]. From a physiological perspective, experimental research has suggested that low doses of alcohol can improve cardiovascular health, lipid profiles, redox status, and the immune system [9,17,18,19,20,21]. In our work, occasional and moderate beer drinkers (women: 1 standard unit of drink per day; men: 2 standard units of drink per day) were more likely to describe better physical health in terms of reduced functional limitations in type and intensity, but also in other relevant components of health that have received less attention, such as perceived health, mental health and socio-emotional domains. 

Certainly, these aspects of health may involve biases, but on the other hand, they may also represent the physical, mental, and socio-emotional health of individuals and how they interact with their daily living conditions as a reflection of their quality of life. Our study is the first to encompass all these factors, as beer consumption shows better indicators of physical, self-perceived, mental, and socio-emotional health in moderate beer drinkers than in abstainers and ex-drinkers. Previous studies in Spain had found that high alcohol consumption was associated with poorer self-perceived health and no differences in socio-affective health [42]. On the other hand, other studies in small samples of populations aged 55 and over showed that moderate consumption of wine, a fermented beverage like beer, was associated with better self-perceived health, mental health and vitality, similar to our results for those aged 40 and over [43]. In Spain, a region with a pattern of alcohol consumption rooted in social gatherings and consumption with other foods compared to northern European countries, could partially and hypothetically explain these differences in mental and socio-affective health between abstainers and ex-drinkers compared to occasional and moderate drinkers. It is noteworthy that these data were also replicated in some Nordic countries, both under and over 40 years of age, which makes the internationalization of the results possible [32,44]. Danish researchers observed in 693 participants aged 29–34 years that simple wine drinking was associated with positive social, cognitive and personality development, while beer consumption reflected some negative outcomes [32]. Furthermore, in Finland, a longitudinal cohort of 2468 people aged 40–55 years indicated that wine consumption reduced cardiovascular mortality and increased quality of life and mental health, but no effect was observed for beer consumption [44]. A sociological study by Sayette et al. in 2012 suggested that, in spontaneous meetings, alcohol consumption, compared to placebo and non-drinking, facilitated the formation and creation of new small groups with casual and strangers meeting each other [45]. They also reported greater positive expressiveness, satisfaction and happiness in creating these new social bonds in the alcohol setting [45]. 

Loneliness and social isolation (stress caused by the discrepancy between actual and desired social relationships) have recently been characterized as a health risk factor, associated with premature mortality and poorer cardiovascular, metabolic, neurological, and mental prognosis [25]. However, we should also mention the possible hidden risks of moderate drinking. Moderate drinking in conjunction with these social meetings could mask and skew consumption towards higher levels of intake. We found that heavy beer drinkers were more likely to report higher self-perceived and mental health, and fewer mild and severe daily limitations. However, habitual and high alcohol consumption could be a health-damaging behavior [16,17,19]. In this respect, the interdependence of different health-related lifestyles should also be taken into account, as health-damaging behaviors can often occur together. In our study, heavy beer drinkers also reported a higher prevalence of tobacco use and a poorer diet, while the groups with the best diet and the lowest tobacco use were abstainers and ex-drinkers. However, the latter two groups also reported the highest prevalence of sedentary behavior. These results alert, on the one hand, to the need for further research on alcohol consumption and, on the other hand, to the interconnectedness and clustering of these behaviors, in which high beer consumption may be a double-edged habit and a health risk factor.

### 4.3. Study Limitations

Despite the potential implications of our findings, the results should be taken with caution due to the several limitations of the study. Firstly, although our study includes two waves of the National Health Survey, representative of the Spanish population, the cross-sectional nature of its data precludes establishing a cause–effect relationship between beer consumption and the subsequent development of a certain health value, as the same participants were not followed longitudinally over time. Secondly, the assessment of health status, alcoholic beer consumption, and some of the health-related lifestyle variables was done through self-reporting by individuals, which may imply under- or overestimation. This type of measurement also involves specific biases associated with memory and recall, mainly on quantitative variables, which are more pronounced in older people and those with very low alcohol consumption [30]. Some types of health-related lifestyle variables may also contain biases related to individual or family socio-economic status and place of residence. For example, behaviors that are socially perceived as harmful may be underestimated by social desirability and subject compliance [30,46]. However, the design of the SNHS, comprising a large sample size representative of the Spanish adult population, allows for a comprehensive assessment of alcohol consumption, such as frequency of alcohol consumption in the last twelve months, frequency of regular weekly drinking from Monday to Sunday in general and specific to different types of alcoholic beverages in number, and in grams of alcohol, in order to establish accurate cut-off points for drinking behaviors. However, in the SNHS, in those drinkers who reported drinking 2–3 times per month, 1 time per month or less than 1 time per month, the exact total alcohol consumption overall and between types of alcoholic beverages was not subsequently assessed. Similarly, beer drinkers were established when their total alcohol consumption came predominantly from beer (more than 50%). In this case, to ensure an adequate sample size for comparisons between groups, we proceeded as follows, defining the groups into non-consumers (abstainers), ex-drinkers (separate from abstainers), and moderate and heavy beer drinkers [30,31,32]. Furthermore, our study only focuses on Spain, so the cultural and social context might influence not only alcohol consumption, but also physical, mental, and social self-perception, which might be different in countries outside the Mediterranean context.

### 4.4. Future Research

Future research should encompass many complementary directions. Special efforts should be devoted to further elucidating the complex relationship of alcohol consumption with health through both physiological and epidemiological research. More evidence is needed not only on general health or mortality, but also with special attention to the mental, social, and emotional domains in relation to light and moderate alcohol consumption in general and beer consumption in particular. Along these lines, different socio-affective domains such as family, friends, partner, work, or leisure could be assessed separately, as well as implementing the measurement of the number and quality of social relationship networks, which in turn may also be closely associated with mental and self-perceived health. Longitudinal studies between alcohol and beer consumption and health outcomes (physical, mental, and socio-affective) would provide strong causal evidence, as would replication of the results in other regions with cultural differences in alcohol consumption, such as in Northern Europe. Therefore, future research should also include multi-country studies to compare possible variations according to country context in relation to beer consumption and subjective perceptions of physical, mental, and social health. In addition, the inclusion of non-alcoholic beer in population-based health surveys could reveal whether the association between moderate consumption and improved health is due to alcoholic or non-alcoholic compounds in beer, which could support non-alcoholic beer as a healthier and more consumer-friendly alternative through public health policies.

## 5. Conclusions

Occasional alcohol drinkers and moderate beer drinkers, compared to abstainers and former drinkers, were more likely to describe better subjective, mental, and socio-affective health, and fewer limitations in daily activity. Heavy beer drinkers were also more likely to describe better self-perceived health, mental health, and fewer mild and severe daily limitations, but showed weaker relationships than moderate beer drinkers. More research is needed in the future, focusing mainly on the socio-affective domain and alcohol consumption to elucidate the complex, and as yet inconclusive, relationship between health and alcohol consumption, despite the association of light to moderate beer consumption with good general health status in the Spanish adult population.

## Figures and Tables

**Table 1:** Socio-demographic and health-related lifestyle characteristics across beer consumption levels (≥18 years), Spanish National Health Surveys 2012, 2017.

	Abstainer	Ex-Drinker	Occasional	Beer Consumption		*p*-Value
				Moderate	High	
**Total (*n,* %)**	8929 (26.91)	6350 (19.14)	11,421 (34.42)	5468 (16.48)	1017 (3.06)	-
**Gender (*n*, %)**						
Men	1956 (21.91)	2393 (37.69)	4735 (41.46)	3673 (67.17)	621 (61.06)	<0.001
Women	6973 (78.09)	3957 (62.31)	6686 (58.54)	1795 (32.83)	396 (38.94)	
**Age (mean, SD)**	58.26 (19.94)	59.95 (18.61)	47.43 (17.24)	46.68 (13.70)	49.27 (12.97)	<0.001
**Age group (*n*, %)**						
18–34	1288 (14.42)	664 (10.46)	2875 (25.17)	1024 (18.73)	132 (12.98)	<0.001
35–64	3787 (42.41)	2858 (45.01)	6433 (56.33)	3846 (70.34)	754 (74.14)	
≥65	3854 (43.16)	2828 (44.54)	2113 (18.50)	598 (10.94)	131 (12.88)	
**Education (*n*, %)**						
Primary or no education	4385 (49.17)	2795 (44.06)	2471 (21.66)	867 (15.86)	221 (21.77)	<0.001
Secondary	3730 (41.83)	2932 (46.22)	6703 (58.76)	3369 (61.65)	611 (60.20)	
University	803 (9.00)	616 (9.71)	2234 (19.58)	1229 (22.49)	183 (18.03)	
**Occupational social class (*n*, %)**						
Low	6009 (72.06)	4341 (70.85)	6698 (60.09)	3042 (56.27)	609 (61.02)	<0.001
Middle	1317 (15.79)	1046 (17.07)	2235 (20.05)	1137 (21.03)	205 (20.54)	
High	1013 (12.15)	740 (12.08)	2214 (19.86)	1227 (22.70)	184 (18.44)	
**Resident place (*n*, %)**						
Rural	2208 (24.73)	1674 (26.36)	2294 (20.09)	1072 (19.60)	273 (26.84)	<0.001
Suburban	5872 (65.76)	3989 (62.82)	7659 (67.06)	3577 (65.42)	607 (59.69)	
Urban	849 (9.51)	687 (10.82)	1468 (12.85)	819 (14.98)	137 (13.47)	
**Year of survey (*n*, %)**						
2012	4558 (51.05)	2839 (44.71)	5131 (44.93)	2492 (45.57)	409 (40.22)	<0.001
2017	4371 (48.95)	3511 (55.29)	6290 (55.07)	2976 (54.43)	608 (59.78)	
**Leisure time physical activity (*n*, %)**						
Never	4738 (53.08)	3213 (50.62)	4285 (37.53)	1953 (35.72)	417 (41.00)	<0.001
Occasionally	3203 (35.88)	2440 (38.44)	4280 (37.48)	1897 (34.69)	366 (35.99)	
Several times per month	495 (5.55)	373 (5.88)	1483 (12.99)	845 (15.45)	136 (13.37)	
Several times per week	490 (5.49)	321 (5.06)	1371 (12.01)	773 (14.14)	98 (9.64)	
**Fruit intake (*n*, %)**						
Almost never or never	204 (2.29)	175 (2.76)	298 (2.61)	211 (3.86)	90 (8.86)	<0.001
Less than once per week	212 (2.38)	177 (2.79)	372 (3.26)	206 (3.77)	74 (7.28)	
Once or twice per week	562 (6.30)	427 (6.73)	1049 (9.19)	578 (10.57)	167 (16.44)	
Three or more times per week, but not daily	1496 (16.77)	1113 (17.54)	2615 (22.90)	1119 (20.46)	219 (21.56)	
Once or more times per day	6448 (72.27)	4453 (70.18)	7086 (62.05)	3354 (61.34)	466 (45.87)	
**Vegetables intake (*n*, %)**						
Almost never or never	125 (1.40)	97 (1.53)	117 (1.02)	50 (0.91)	19 (1.87)	<0.001
Less than once per week	205 (2.30)	142 (2.24)	253 (2.22)	136 (2.49)	34 (3.35)	
Once or twice per week	1012 (11.35)	692 (10.91)	1265 (11.08)	659 (12.05)	139 (13.69)	
Three or more times per week, but not daily	3546 (39.77)	2625 (41.39)	4816 (42.18)	2278 (41.66)	443 (43.65)	
Once or more times per day	4029 (45.18)	2786 (43.93)	4967 (43.50)	2345 (42.89)	380 (37.44)	
**Sweets intake (*n*, %)**						
Almost never or never	1729 (19.40)	1241 (19.57)	1613 (14.13)	873 (15.97)	214 (21.10)	<0.001
Less than once per week	1469 (16.48)	1119 (17.65)	1947 (17.06)	889 (16.26)	182 (17.95)	
Once or twice per week	1650 (18.51)	1166 (18.39)	2543 (22.28)	1219 (22.30)	217 (21.40)	
Three or more times per week, but not daily	1548 (17.37)	1012 (15.96)	2249 (19.70)	1062 (19.43)	184 (18.15)	
Once or more times per day	2516 (28.23)	3063 (26.83)	3063 (26.83)	1423 (26.03)	217 (21.40)	
**Sweetened beverages intake (*n*, %)**						
Almost never or never	5173 (58.06)	3538 (55.85)	4900 (42.95)	2289 (41.91)	454 (44.73)	<0.001
Less than once per week	1333 (14.96)	1152 (18.18)	2385 (20.90)	1089 (19.94)	207 (20.39)	
Once or twice per week	1071 (12.02)	693 (10.94)	1952 (17.11)	961 (17.59)	142 (13.99)	
Three or more times per week, but not daily	605 (6.79)	412 (6.50)	1055 (9.25)	566 (10.36)	86 (8.47)	
Once or more times per day	728 (8.17)	540 (8.52)	1117 (9.79)	557 (10.20)	126 (12.41)	
**Fast food intake (*n*, %)**						
Almost never or never	5236 (58.79)	3538 (55.84)	4392 (38.49)	1884 (34.48)	346 (34.16)	<0.001
Less than once per week	1998 (22.43)	1596 (25.19)	3493 (30.61)	1663 (30.44)	305 (30.11)	
Once or twice per week	1309 (14.70)	942 (14.87)	2820 (24.72)	1517 (27.76)	275 (27.15)	
Three or more times per week, but not daily	304 (3.41)	205 (3.24)	568 (4.98)	299 (5.47)	60 (5.92)	
Once or more times per day	59 (0.66)	55 (0.87)	137 (1.20)	101 (1.85)	27 (2.67)	
**Snacks intake (*n*, %)**						
Almost never or never	4905 (55.06)	3430 (54.12)	4501 (39.46)	1734 (31.73)	288 (28.37)	<0.001
Less than once per week	2315 (25.99)	1713 (27.03)	3531 (30.95)	1728 (31.63)	288 (28.37)	
Once or twice per week	1229 (13.80)	926 (14.61)	2647 (23.21)	1504 (27.53)	273 (26.90)	
Three or more times per week, but not daily	379 (4.25)	219 (3.46)	601 (5.27)	397 (7.27)	127 (12.51)	
Once or more times per day	80 (0.90)	50 (0.79)	127 (1.11)	101 (1.85)	39 (3.84)	
**Body Mass Index (*n*, %)**						
Insufficient weight	176 (2.22)	108 (1.89)	252 (2.33)	71 (1.34)	23 (2.33)	<0.001
Normal weight	3221 (40.57)	2195 (38.43)	5043 (46.65)	2355 (44.29)	425 (43.06)	
Overweight	2834 (35.70)	2164 (37.89)	3784 (35.00)	2145 (40.34)	379 (38.40)	
Obesity	1708 (21.51)	1244 (21.78)	1731 (16.01)	746 (14.03)	160 (16.21)	
**Tobacco use (*n*, %)**						
Never	6892 (77.23)	3513 (55.36)	5930 (51.94)	1939 (35.60)	188 (18.50)	<0.001
Ex-smoker	906 (10.15)	1619 (25.51)	2528 (22.14)	1527 (27.93)	248 (24.41)	
Current smoker	1126 (12.62)	1214 (19.13)	2960 (25.92)	2001 (36.60)	580 (57.09)	

## Data Availability

Statistical analysis code is available at GitHub (https://github.com/antoniomoreno13/Functional-benefits-of-moderate-consumption-of-beer-in-the-Spanish-adult-population, accessed on 4 January 2023).

## Supplementary Materials

The following supporting information can be downloaded at https://www.mdpi.com/article/10.3390/nu15061519/s1. Table S1: General characteristics of the sample (≥18 years) of the Spanish National Health Surveys 2012, 2017; Figure S1: Odds Ratio and 95% Confidence Intervals of reporting good self-perceived health in the Spanish adult population (≥18 years) in those who never had an acute heavy drinking episode according to beer consumption; Figure S2: Odds Ratio and 95% Confidence Intervals of reporting good self-perceived health in the Spanish adult population (≥18 years) by gender according to beer consumption; Figure S3: Odds Ratio and 95% Confidence Intervals of reporting good self-perceived health in the Spanish adult population (≥18 years) by age groups according to beer consumption; Figure S4: Odds Ratio and 95% Confidence Intervals of reporting daily physical, mental or both limitations in the Spanish adult population (≥18 years) in those who never had an acute heavy drinking episode according to beer consumption; Figure S5: Odds Ratio and 95% Confidence Intervals of reporting daily physical, mental or both limitations in the Spanish adult population (≥18 years) by gender according to beer consumption; Figure S6: Odds Ratio and 95% Confidence Intervals of reporting daily physical, mental or both limitations in the Spanish adult population (≥18 years) by age groups according to beer consumption; Figure S7: Odds Ratio and 95% Confidence Intervals of reporting daily mild or severe limitations in the Spanish adult population (≥18 years) in those who never had an acute heavy drinking episode according to beer consumption; Figure S8: Odds Ratio and 95% Confidence Intervals of reporting daily mild or severe limitations in the Spanish adult population (≥18 years) by gender according to beer consumption; Figure S9: Odds Ratio and 95% Confidence Intervals of reporting daily mild or severe limitations in the Spanish adult population (≥18 years) by age groups according to beer consumption; Figure S10: Odds Ratio and 95% Confidence Intervals of reporting medium and good mental health in the Spanish adult population (≥18 years) in those who never had an acute heavy drinking episode according to beer consumption; Figure S11: Odds Ratio and 95% Confidence Intervals of reporting medium and good mental health in the Spanish adult population (≥18 years) by gender according to beer consumption; Figure S12: Odds Ratio and 95% Confidence Intervals of reporting medium and good mental health in the Spanish adult population (≥18 years) by age groups according to beer consumption; Figure S13: Odds Ratio and 95% Confidence Intervals of reporting medium and good social-affective support in the Spanish adult population (≥18 years) in those who never had an acute heavy drinking episode according to beer consumption; Figure S14: Odds Ratio and 95% Confidence Intervals of reporting medium and good social-affective support in the Spanish adult population (≥18 years) by gender according to beer consumption; Figure S15: Odds Ratio and 95% Confidence Intervals of reporting medium and good social-affective support in the Spanish adult population (≥18 years) by age groups according to beer consumption.
