# Supplementary material for: Moderate Beer Consumption Is Associated with Good Physical and Mental Health Status and Increased Social Support
Source: Nutrients. 2023 Mar 21;15(6):1519. doi: 10.3390/nu15061519 (PMC10052738; doi:10.3390/nu15061519)
Supplement: Supplementary file 1 [file nutrients-15-01519-s001.zip › nutrients-2269385-supplementary.pdf]

Supplementary Material for:

Moderate beer consumption is associated good physical and mental health status and higher social support

This file includes:

Table S1

Figures S1 to S15

Table S1. General characteristics of the sample (≥18 years) of the Spanish National Health Surveys 2012, 2017

**Total (*n*, %)**	43098 (100)
**Gender (*n*, %)**	
Men	19716 (45.75)
Women	23382 (54.25)
**Age (mean, SD)**	53.41 (18.39)
**Age group (*n*, %)**	
18-34	7309 (16.96)
35-64	22870 (53.06)
≥65	12919 (29.98)
**Education (*n*, %)**	
Primary or no education	13684 (31.79)
Secondary	22316 (51.83)
University	7053 (16.38)
**Occupational social class (*n*, %)**	
Low	26312 (63.02)
Middle	7860 (18.82)
High	7582 (18.16)
**Resident place (*n*, %)**	
Rural	10165 (23.59)
Suburban	27803 (64.51)
Urban	5130 (11.90)
**Year of survey (*n*, %)**	
2012	20587 (47.77)
2017	22511 (52.23)
**Leisure time physical activity (*n*, %)**	
Never	18002 (41.79)
Occasionally	16414 (38.11)
Several times per month	4630 (10.75)
Several times per week	4026 (9.35)
**Fruit intake (*n*, %)**	
Almost never or never	1260 (2.93)
Less than once per week	1377 (3.20)
Once or twice per week	3618 (8.40)
Three or more times per week, but not daily	8255 (19.17)
Once or more times per day	28549 (66.30)
**Vegetables intake (*n*, %)**	
Almost never or never	521 (1.21)
Less than once per week	981 (2.28)
Once or twice per week	4776 (11.10)
Three or more times per week, but not daily	17522 (40.70)
Once or more times per day	19248 (44.71)
**Sweets intake (*n*, %)**	
Almost never or never	7475 (17.38)
Less than once per week	7419 (17.24)
Once or twice per week	8687 (20.19)
Three or more times per week, but not daily	7556 (17.56)

Table S1. Continued

Once or more times per day	11885 (27.63)
**Sweetened beverages intake (*n*, %)**	
Almost never or never	21665 (50.39)
Less than once per week	8103 (18.84)
Once or twice per week	6052 (14.08)
Three or more times per week, but not daily	3407 (7.92)
Once or more times per day	3772 (8.77)
**Fast food intake (*n*, %)**	
Almost never or never	20391 (47.42)
Less than once per week	11727 (27.27)
Once or twice per week	8568 (19.93)
Three or more times per week, but not daily	1795 (4.17)
Once or more times per day	521 (1.21)
**Snacks intake (*n*, %)**	
Almost never or never	19293 (44.86)
Less than once per week	12344 (28.70)
Once or twice per week	8538 (19.85)
Three or more times per week, but not daily	2285 (5.32)
Once or more times per day	547 (1.27)
**Body Mass Index (*n*, %)**	
Insufficient weight	750 (1.87)
Normal weight	16819 (41.89)
Overweight	15353 (38.23)
Obesity	7231 (18.01)
**Tobacco use (*n*, %)**	
Never	22313 (51.83)
Ex-smoker	10120 (23.50)
Current smoker	10620 (24.67)
**Self-rated health status (*n*, %)**	
Good	28670 (66.52)
Poor	14428 (33.48)
**Type of limitations (*n*, %)**	
Physical	9558 (22.18)
Mental	590 (1.37)
Both	1248 (2.90)
None	31685 (73.55)
**Intensity of limitations (*n*, %)**	
None	31685 (73.54)
Mild	9221 (21.40)
Severe	2181 (5.06)
**Mental health terciles (*n*, %)**	
Good	35605 (85.34)
Medium	4479 (10.74)
Poor	1638 (3.92)

Table S1. Continued

**Social support terciles (*n*, %)**	
Good	34862 (85.09)
Medium	5503 (13.43)
Poor	608 (1.42)
**Abusive alcohol consumption (*n*, %)**	
Never	23061 (53.68)
Not in the last 12 months	6350 (14.78)
Less than once a month	11085 (25.80)
Monthly	947 (2.20)
Weekly	1381 (3.21)
Daily or almost daily	138 (0.32)
**Alcohol consumption (*n*, %)**	
Abstainer	8929 (20.87)
Ex-drinker	6350 (14.84)
Occasional	11421 (26.69)
Beer moderate	5468 (12.78)
Beer high	1017 (2.38)
Wine moderate	4756 (11.12)
Wine high	1010 (2.36)
Spirits moderate	844 (1.97)
Spirits high	272 (0.64)
Vermouth moderate	78 (0.18)
Vermouth high	42 (0.10)
Liquors moderate	91 (0.21)
Liquors high	47 (0.11)
Locals moderate	124 (0.29)
Locals high	53 (0.12)
Mixed moderate	1741 (4.07)
Mixed high	544 (1.27)
**Type of drinker (*n*, %)**	
Abstainer	8929 (20.87)
Ex-drinker	6350 (14.84)
Occasional	11421 (26.69)
Beer	6485 (15.16)
Wine	5766 (13.48)
Spirits	1116 (2.61)
Vermouth	120 (0.28)
Liquors	138 (0.32)
Locals	177 (0.41)
Mixed	2285 (5.34)
**Alcohol consumption intensity (*n*, %)**	
Abstainer	8929 (20.89)
Ex-drinker	6350 (14.84)
Occasional	11421 (26.69)
Moderate	13102 (30.62)
High	2985 (6.98)

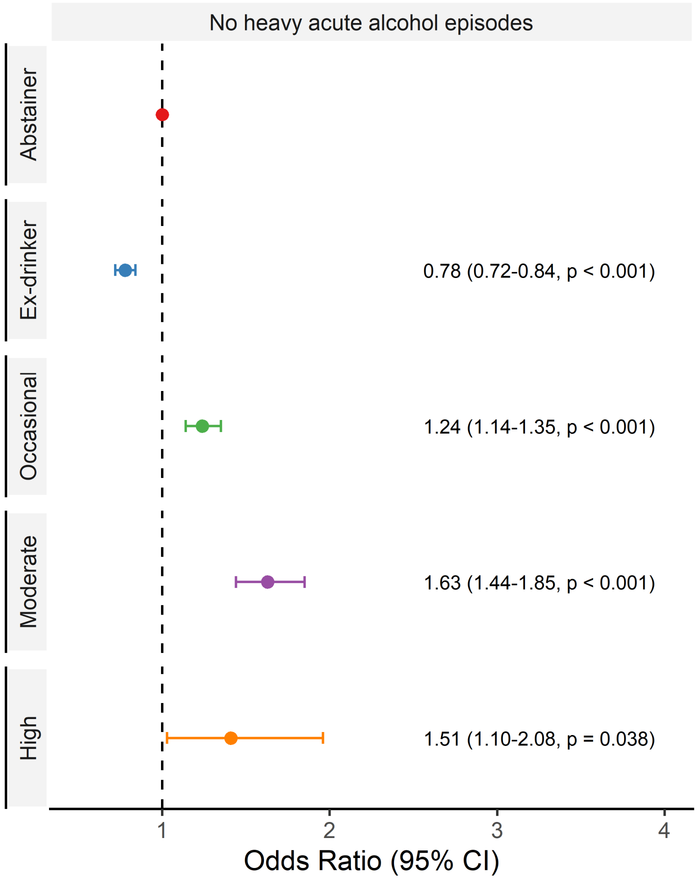

Figure S1. Odds Ratio and 95% Confidence Intervals of reporting good self-perceived health in the Spanish adult population (≥18 years) in those who never had an acute heavy drinking episode according to beer consumption. Drinkers were classified as occasional (a maximum of 3 days per month), moderate (Women: up to 12 g of alcohol per day, Men: up to 24 g of alcohol per day) or heavy (Women: more than 12 g of alcohol per day, Men: more than 24 g of alcohol per day). Analyses adjusted for age, gender, social class, educational level, year of the survey, place of residence, physical activity, diet, tobacco, and body mass index. National Health Survey of Spain 2012, 2017.

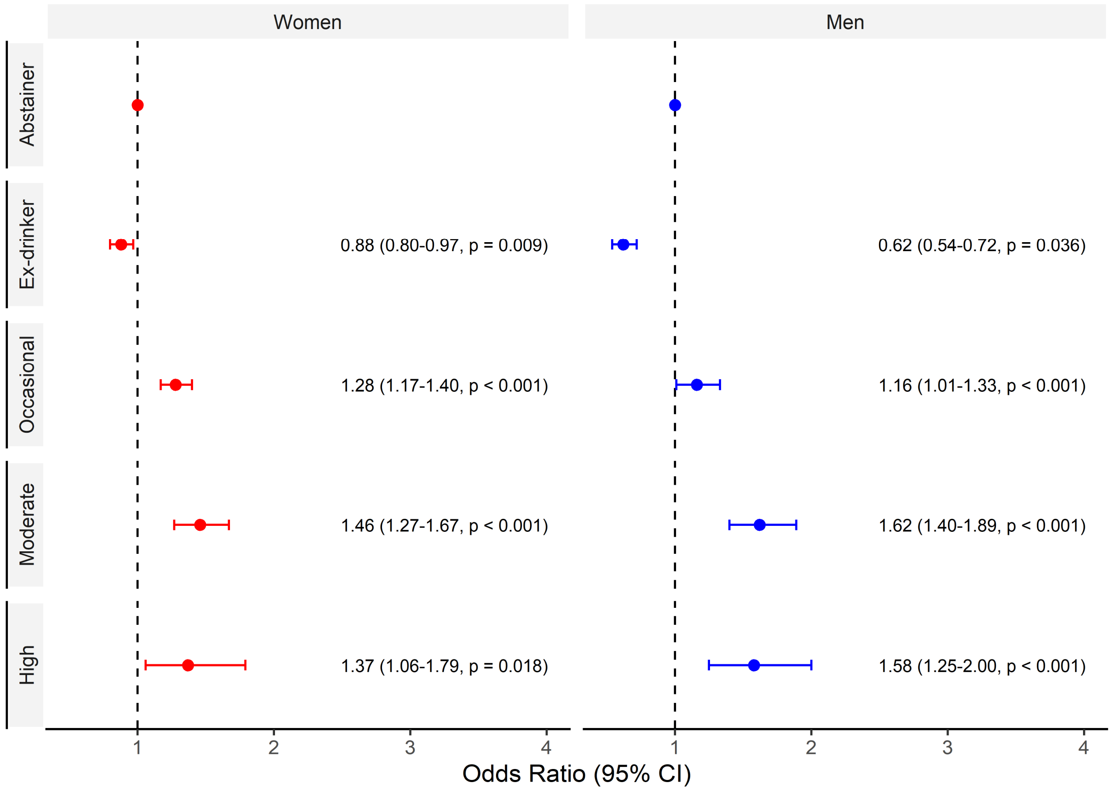

Figure S2. Odds Ratio and 95% Confidence Intervals of reporting good self-perceived health in the Spanish adult population (≥18 years) by gender according to beer consumption. Drinkers were classified as occasional (a maximum of 3 days per month), moderate (Women: up to 12 g of alcohol per day, Men: up to 24 g of alcohol per day) or high (Women: more than 12 g of alcohol per day, Men: more than 24 g of alcohol per day). Analyses adjusted for age, gender, social class, educational level, year of the survey, place of residence, physical activity, diet, tobacco, and body mass index. National Health Survey of Spain 2012, 2017.

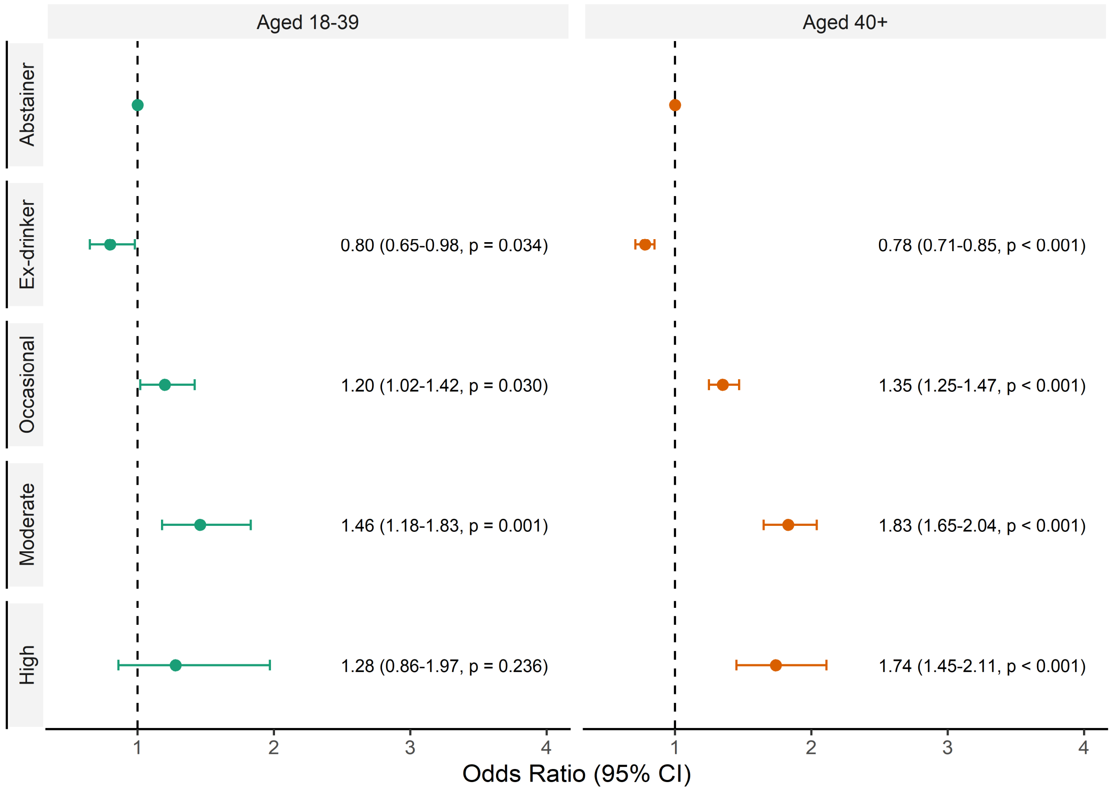

Figure S3. Odds Ratio and 95% Confidence Intervals of reporting good self-perceived health in the Spanish adult population (≥18 years) by age groups according to beer consumption. Drinkers were classified as occasional (a maximum of 3 days per month), moderate (Women: up to 12 g of alcohol per day, Men: up to 24 g of alcohol per day) or high (Women: more than 12 g of alcohol per day, Men: more than 24 g of alcohol per day). Analyses adjusted for age, gender, social class, educational level, year of the survey, place of residence, physical activity, diet, tobacco, and body mass index. National Health Survey of Spain 2012, 2017.

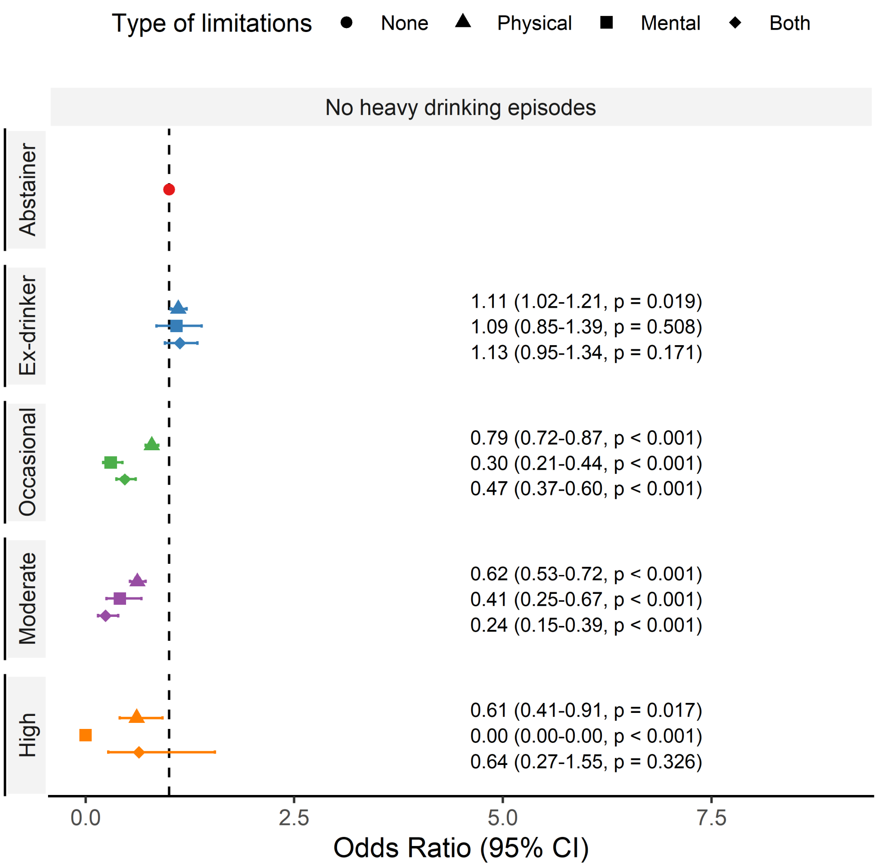

Figure S4. Odds Ratio and 95% Confidence Intervals of reporting daily physical, mental or both limitations in the Spanish adult population (≥18 years) in those who never had an acute heavy drinking episode according to beer consumption. Drinkers were classified as occasional (a maximum of 3 days per month), moderate (Women: up to 12 g of alcohol per day, Men: up to 24 g of alcohol per day) or high (Women: more than 12 g of alcohol per day, Men: more than 24 g of alcohol per day). Analyses adjusted for age, gender, social class, educational level, year of the survey, place of residence, physical activity, diet, tobacco, and body mass index. National Health Survey of Spain 2012, 2017.

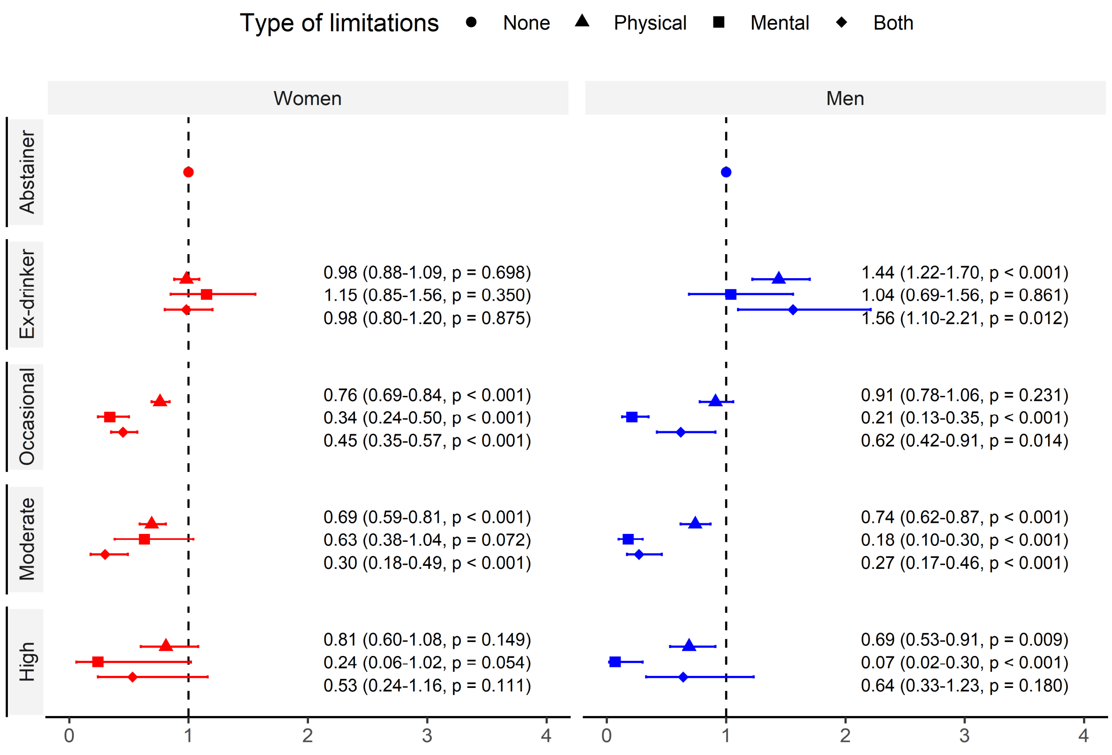

Figure S5. Odds Ratio and 95% Confidence Intervals of reporting daily physical, mental or both limitations in the Spanish adult population (≥18 years) by gender according to beer consumption. Drinkers were classified as occasional (a maximum of 3 days per month), moderate (Women: up to 12 g of alcohol per day, Men: up to 24 g of alcohol per day) or high (Women: more than 12 g of alcohol per day, Men: more than 24 g of alcohol per day). Analyses adjusted for age, gender, social class, educational level, year of the survey, place of residence, physical activity, diet, tobacco, and body mass index. National Health Survey of Spain 2012, 2017.

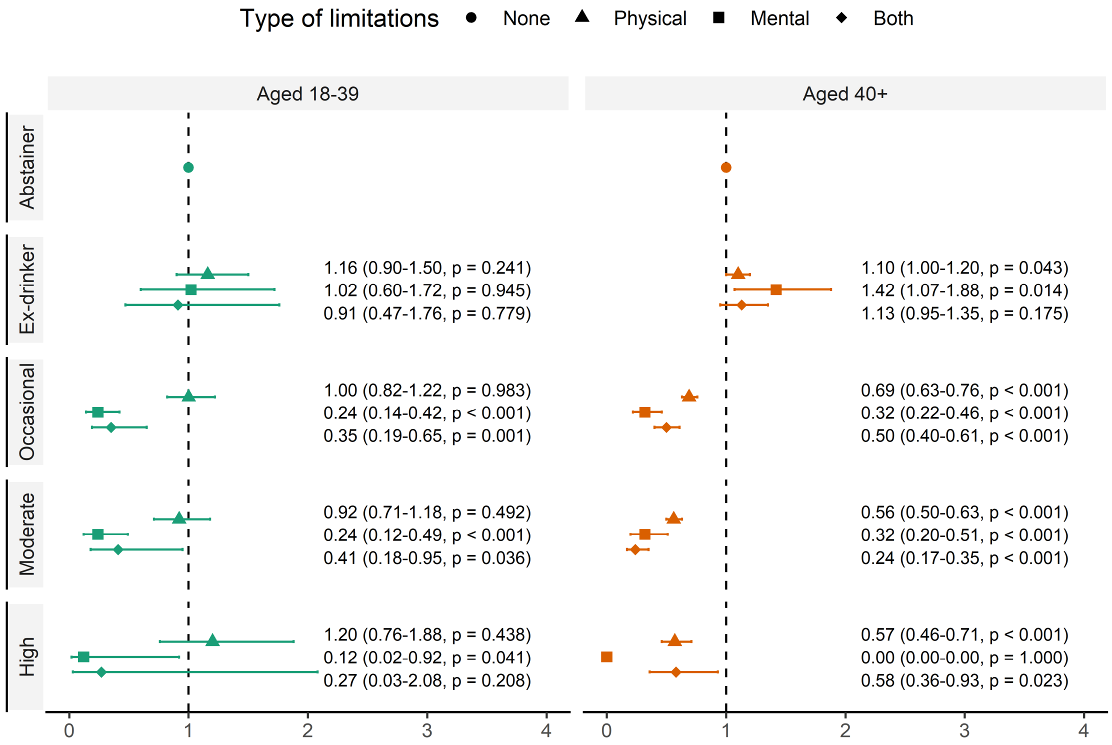

Figure S6. Odds Ratio and 95% Confidence Intervals of reporting daily physical, mental or both limitations in the Spanish adult population (≥18 years) by age groups according to beer consumption. Drinkers were classified as occasional (a maximum of 3 days per month), moderate (Women: up to 12 g of alcohol per day, Men: up to 24 g of alcohol per day) or high (Women: more than 12 g of alcohol per day, Men: more than 24 g of alcohol per day). Analyses adjusted for age, gender, social class, educational level, year of the survey, place of residence, physical activity, diet, tobacco, and body mass index. National Health Survey of Spain 2012, 2017.

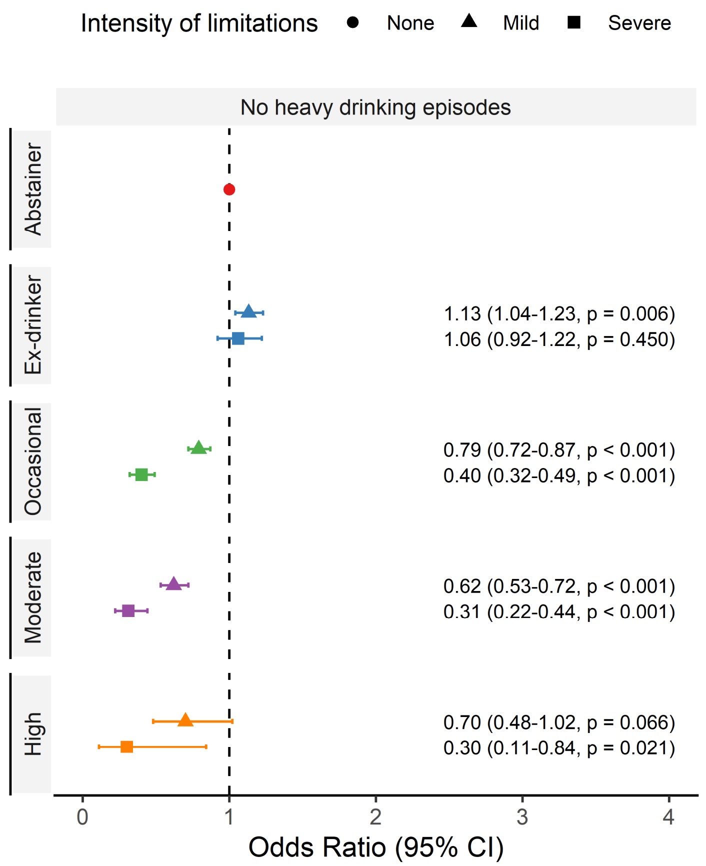

Figure S7. Odds Ratio and 95% Confidence Intervals of reporting daily mild or severe limitations in the Spanish adult population (≥18 years) in those who never had an acute heavy drinking episode according to beer consumption. Drinkers were classified as occasional (a maximum of 3 days per month), moderate (Women: up to 12 g of alcohol per day, Men: up to 24 g of alcohol per day) or high (Women: more than 12 g of alcohol per day, Men: more than 24 g of alcohol per day). Analyses adjusted for age, gender, social class, educational level, year of the survey, place of residence, physical activity, diet, tobacco, and body mass index. National Health Survey of Spain 2012, 2017.

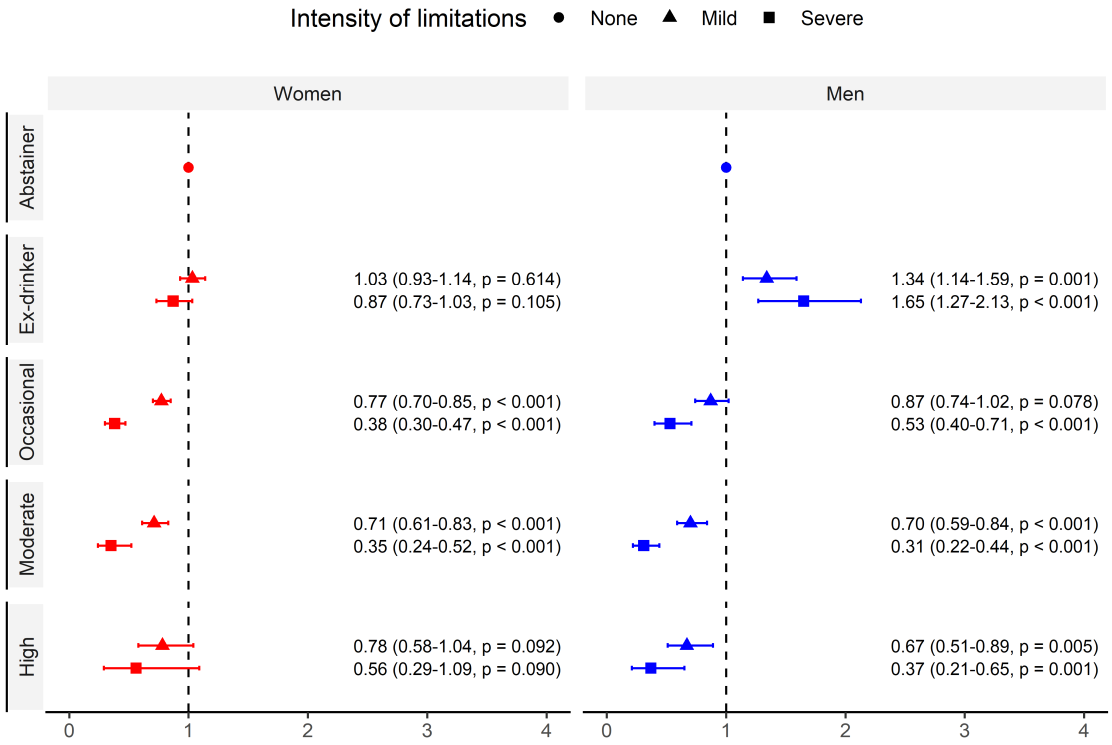

Figure S8. Odds Ratio and 95% Confidence Intervals of reporting daily mild or severe limitations in the Spanish adult population (≥18 years) by gender according to beer consumption. Drinkers were classified as occasional (a maximum of 3 days per month), moderate (Women: up to 12 g of alcohol per day, Men: up to 24 g of alcohol per day) or high (Women: more than 12 g of alcohol per day, Men: more than 24 g of alcohol per day). Analyses adjusted for age, gender, social class, educational level, year of the survey, place of residence, physical activity, diet, tobacco, and body mass index. National Health Survey of Spain 2012, 2017.

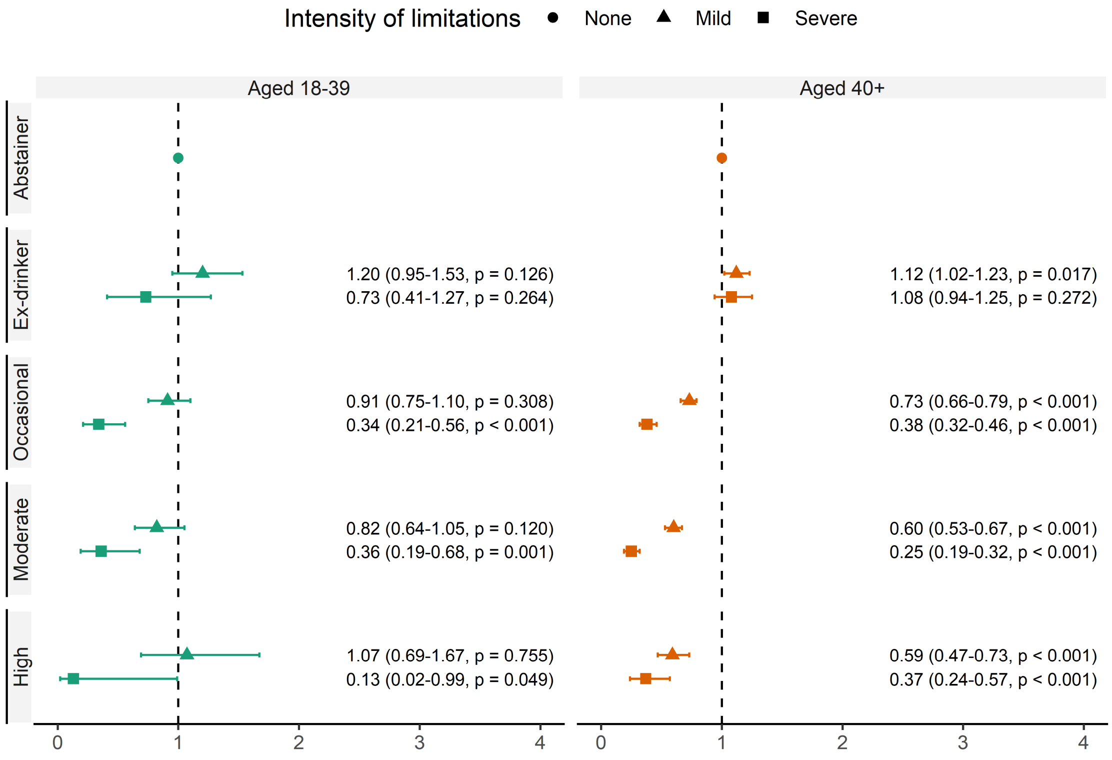

Figure S9. Odds Ratio and 95% Confidence Intervals of reporting daily mild or severe limitations in the Spanish adult population (≥18 years) by age groups according to beer consumption. Drinkers were classified as occasional (a maximum of 3 days per month), moderate (Women: up to 12 g of alcohol per day, Men: up to 24 g of alcohol per day) or high (Women: more than 12 g of alcohol per day, Men: more than 24 g of alcohol per day). Analyses adjusted for age, gender, social class, educational level, year of the survey, place of residence, physical activity, diet, tobacco, and body mass index. National Health Survey of Spain 2012, 2017.

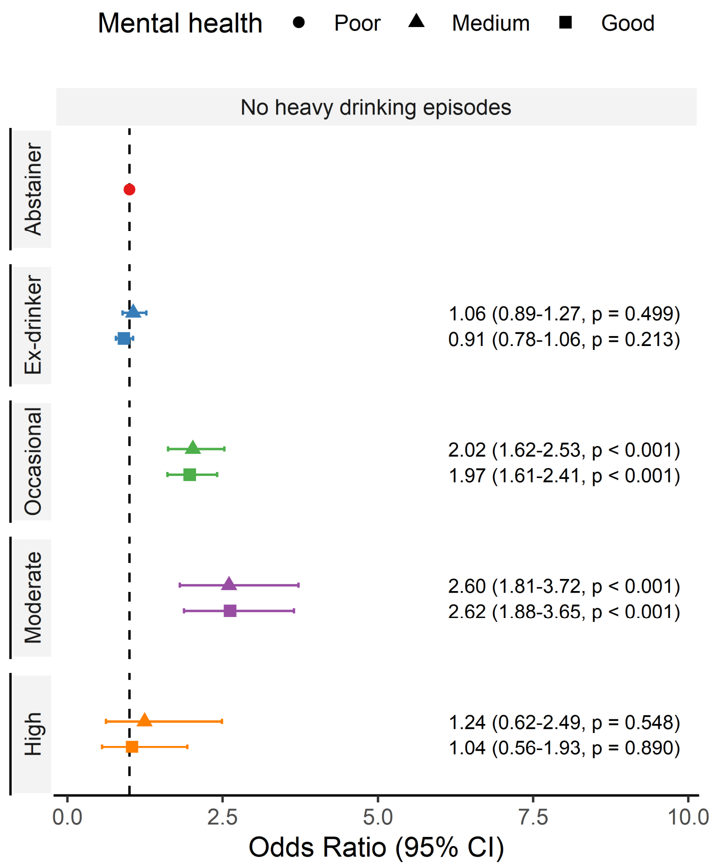

Figure S10. Odds Ratio and 95% Confidence Intervals of reporting medium and good mental health in the Spanish adult population (≥18 years) in those who never had an acute heavy drinking episode according to beer consumption. Drinkers were classified as occasional (a maximum of 3 days per month), moderate (Women: up to 12 g of alcohol per day, Men: up to 24 g of alcohol per day) or high (Women: more than 12 g of alcohol per day, Men: more than 24 g of alcohol per day). Analyses adjusted for age, gender, social class, educational level, year of the survey, place of residence, physical activity, diet, tobacco, and body mass index. National Health Survey of Spain 2012, 2017.

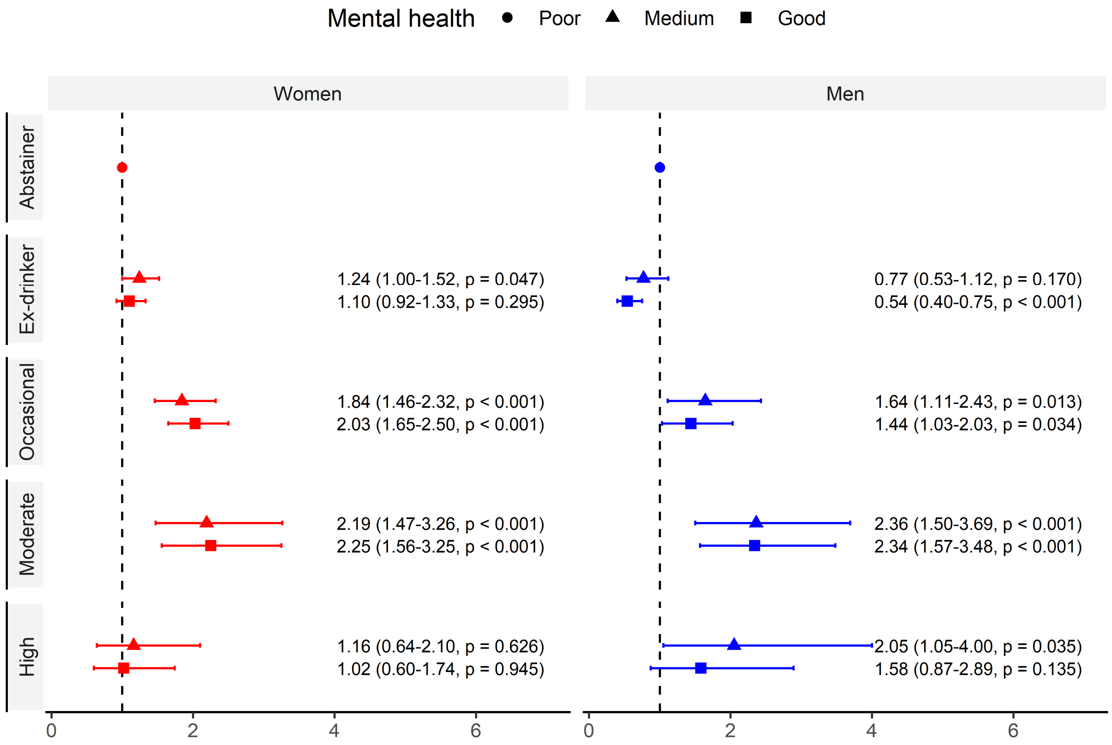

Figure S11. Odds Ratio and 95% Confidence Intervals of reporting medium and good mental health in the Spanish adult population (≥18 years) by gender according to beer consumption. Drinkers were classified as occasional (a maximum of 3 days per month), moderate (Women: up to 12 g of alcohol per day, Men: up to 24 g of alcohol per day) or high (Women: more than 12 g of alcohol per day, Men: more than 24 g of alcohol per day). Analyses adjusted for age, gender, social class, educational level, year of the survey, place of residence, physical activity, diet, tobacco, and body mass index. National Health Survey of Spain 2012, 2017.

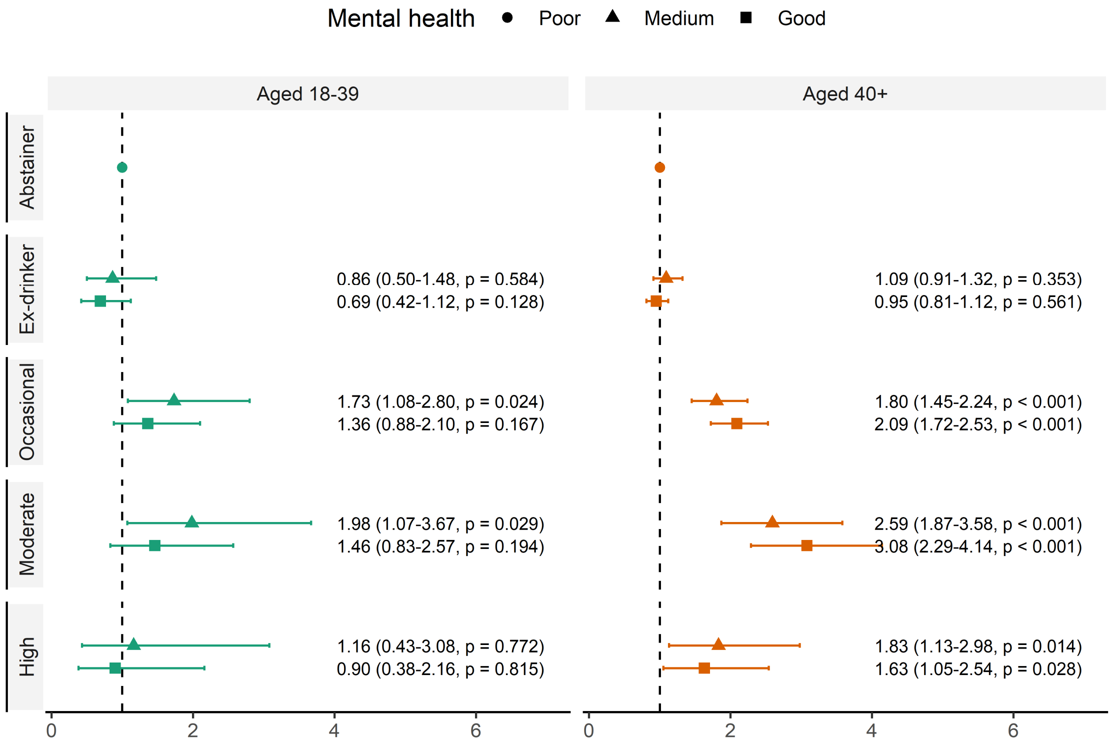

Figure S12. Odds Ratio and 95% Confidence Intervals of reporting medium and good mental health in the Spanish adult population (≥18 years) by age groups according to beer consumption. Drinkers were classified as occasional (a maximum of 3 days per month), moderate (Women: up to 12 g of alcohol per day, Men: up to 24 g of alcohol per day) or high (Women: more than 12 g of alcohol per day, Men: more than 24 g of alcohol per day). Analyses adjusted for age, gender, social class, educational level, year of the survey, place of residence, physical activity, diet, tobacco, and body mass index. National Health Survey of Spain 2012, 2017.

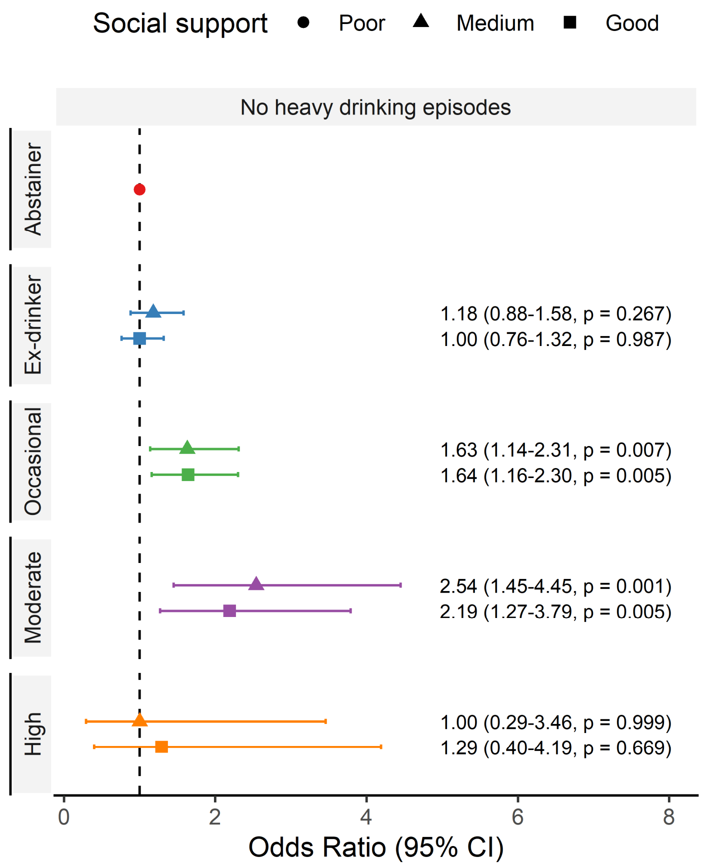

Figure S13. Odds Ratio and 95% Confidence Intervals of reporting medium and good social-affective support in the Spanish adult population (≥18 years) in those who never had an acute heavy drinking episode according to beer consumption. Drinkers were classified as occasional (a maximum of 3 days per month), moderate (Women: up to 12 g of alcohol per day, Men: up to 24 g of alcohol per day) or high (Women: more than 12 g of alcohol per day, Men: more than 24 g of alcohol per day). Analyses adjusted for age, gender, social class, educational level, year of the survey, place of residence, physical activity, diet, tobacco, and body mass index. National Health Survey of Spain 2012, 2017.

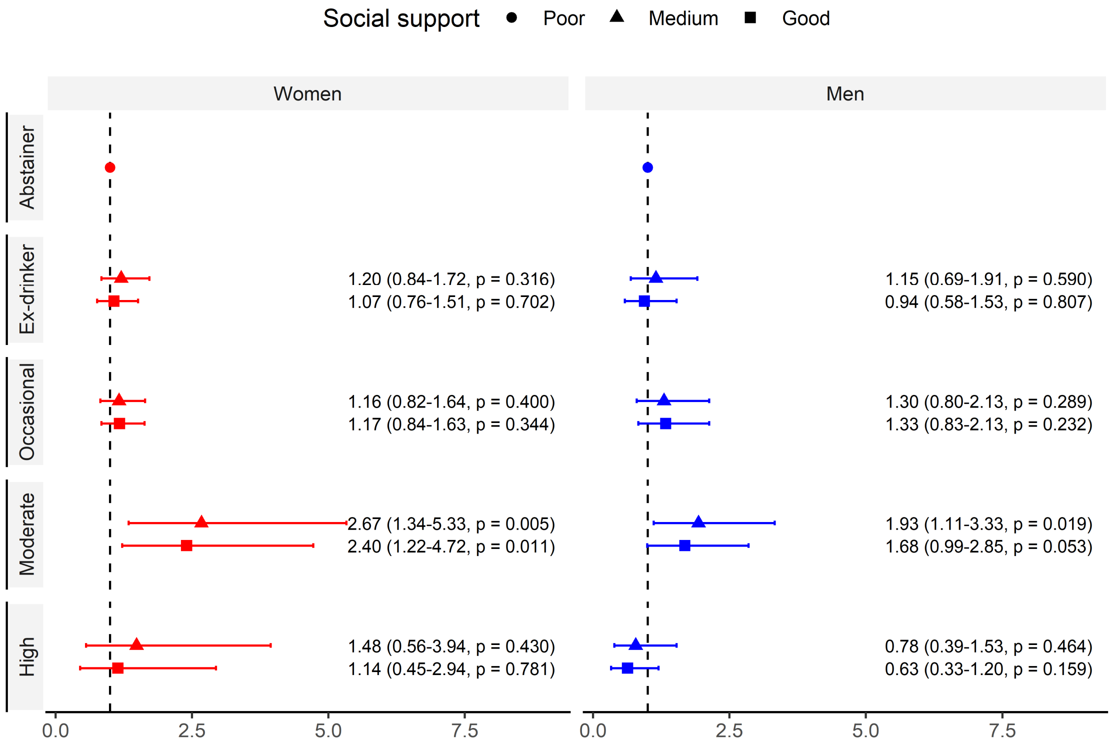

Figure S14. Odds Ratio and 95% Confidence Intervals of reporting medium and good social-affective support in the Spanish adult population (≥18 years) by gender according to beer consumption. Drinkers were classified as occasional (a maximum of 3 days per month), moderate (Women: up to 12 g of alcohol per day, Men: up to 24 g of alcohol per day) or high (Women: more than 12 g of alcohol per day, Men: more than 24 g of alcohol per day). Analyses adjusted for age, gender, social class, educational level, year of the survey, place of residence, physical activity, diet, tobacco, and body mass index. National Health Survey of Spain 2012, 2017.

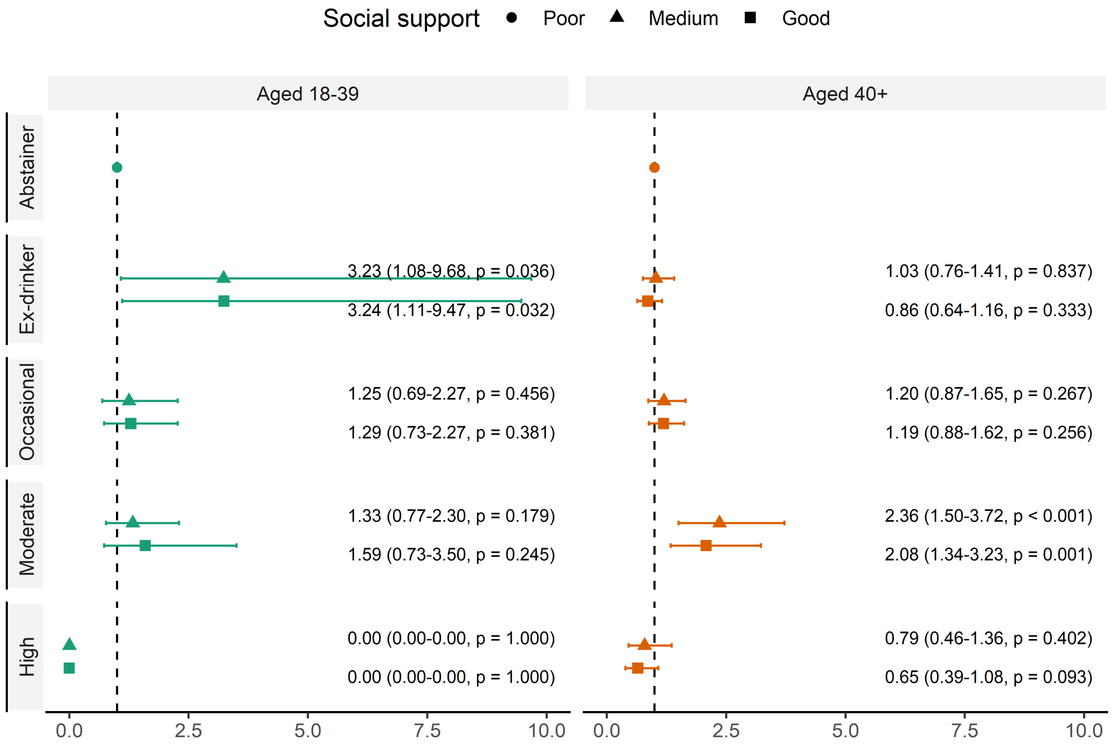

Figure S15. Odds Ratio and 95% Confidence Intervals of reporting medium and good social-affective support in the Spanish adult population (≥18 years) by age groups according to beer consumption. Drinkers were classified as occasional (a maximum of 3 days per month), moderate (Women: up to 12 g of alcohol per day, Men: up to 24 g of alcohol per day) or high (Women: more than 12 g of alcohol per day, Men: more than 24 g of alcohol per day). Analyses adjusted for age, gender, social class, educational level, year of the survey, place of residence, physical activity, diet, tobacco, and body mass index. National Health Survey of Spain 2012, 2017.
